# Exploring gender and thematic differences in qualitative assessments of internal medicine resident performance

**DOI:** 10.1186/s12909-023-04917-7

**Published:** 2023-12-08

**Authors:** Robin Klein, Erin D. Snyder, Jennifer Koch, Anna Volerman, Sarah Alba-Nguyen, Katherine A Julian, Vanessa Thompson, Nneka N Ufere, Sherri-Ann M Burnett-Bowie, Anshul Kumar, Bobbie Ann Adair White, Yoon Soo Park, Kerri Palamara

**Affiliations:** 1 Emory University School of Medicine, Atlanta, GA USA; 2 Department of Medicine, Division of General Internal Medicine, University of Alabama Birmingham School of Medicine, Birmingham, AL USA; 3 Department of Medicine, University of Louisville, Louisville, KY USA; 4 Departments of Medicine and Pediatrics, University of Chicago, Chicago, IL USA; 5 Department of Medicine, Division of Hospital Medicine, University of California, San Francisco, San Francisco, CA USA; 6 Department of Medicine, Division of General Internal Medicine, University of California, San Francisco, San Francisco, CA USA; 7 Department of Medicine, Division of Gastroenterology, Massachusetts General Hospital, Boston, MA USA; 8 Department of Medicine, Endocrine Division, Massachusetts General Hospital, Boston, MA USA; 9 Massachusetts General Hospital Institute of Health Professions, Boston, MA USA; 10 Department of Medical Education, University of Illinois, Chicago, IL USA; 11 Department of Medicine, Massachusetts General Hospital, Boston, MA USA

**Keywords:** Assessment, Graduate medical education, Gender, Qualitative, Internal medicine, Bias, Resident

## Abstract

**Introduction:**

Evidence suggests gender disparities in medical education assessment, including differences in ratings of competency and narrative comments provided in resident performance assessments. This study explores how gender manifests within the content of qualitative assessments (i.e., narrative comments or performance feedback) of resident performance.

**Methods:**

Qualitative content analysis was used to explore gender-based differences in narrative comments included in faculty assessments of resident performance during inpatient medicine rotations at six Internal Medicine residency programs, 2016–2017. A blinded, multi-analyst approach was employed to identify themes across comments. Patterns in themes with resident gender and post-graduate year (PGY) were explored, focusing on PGY2 and PGY3 when residents are serving in the team leader role.

**Results:**

Data included 3,383 evaluations with narrative comments of 385 men (55.2%) and 313 women residents (44.8%). There were thematic differences in narrative comments received by men and women residents and how these themes manifested within comments changed with training time. Compared to men, comments about women had a persistent relationship-orientation and emphasized confidence over training including as interns and in PGY2 and PGY3, when serving as team leader. The relationship-orientation was characterized not only by the residents’ communal attributes but also their interpersonal and communication skills, including efforts supporting others and establishing the tone for the team. Comments about women residents often highlighted confidence, including recommendations around behaviors that convey confidence in decision-making and team leadership.

**Discussion:**

There were gender-based thematic differences in qualitative assessments. Comments about women resident team leaders highlight relationship building skills and urge confidence and actions that convey confidence as team leader. Persistent attention to communal skills suggests gendered expectations for women resident team leaders and a lost opportunity for well-rounded feedback to the disadvantage of women residents. These findings may inform interventions to promote equitable assessment, such as providing feedback across the competencies.

## Introduction

Inequities associated with gender may threaten the integrity of assessment in medical education [1, 2]. Evidence suggests differences associated with gender in faculty assessments of learner performance, including in both competency ratings and narrative comments provided to men and women [2–12].

Studies have reported differences in the traits ascribed to learners in Medical Student Performance Evaluations, clerkship assessments, and clinical performance assessments of residents [5–10]. Women are more often described using relationship-oriented or communal terms such as “compassionate” or “caring” while men are more often described using terms such as “intelligent” or “leader” [8–10].

A recent study of narrative comments included in clinical performance assessments of Internal Medicine (IM) residents found that women residents receive more positively toned but less specific comments than men residents suggesting that comments of women may contain more rote praise but lacking insight [11]. Other studies have suggested women received more discordant feedback across faculty members and that feedback often references resident confidence [12–14]. What remains unclear is whether and how the content differs, specifically the skills and behaviors that are highlighted, in qualitative assessments of men and women residents.

Understanding how qualitative assessments may differ between men and women residents has important implications as these comments serve as critical feedback about learner performance to both learners and programs. This understanding may be translated into opportunities for faculty development. This study aims to explore how gender manifests within narrative assessment of IM residents and to identify thematic differences in comments provided to men and women residents.

## Methods

We performed a retrospective, cross-sectional study of narrative comments included in faculty assessments of IM resident performance during inpatient medicine rotations from six U.S. IM residency training programs, July 2016 to June 2017.

We applied qualitative content analysis, a systematic means to describe patterns of meaning within textual data, to explore gender differences in narrative comments included in resident assessments [15, 16].

### Data

Data was collected from faculty assessments of IM resident performance during general medicine inpatient rotations from the 2016–2017 academic year at six US IM residency training programs.

In the United States, medical education and training involves 3 or more years of post-graduate residency training following medical school. IM residency training involves clinical rotations during which trainees provide care for patients under the supervision of faculty. Data included assessments of residents from inpatient general medicine rotations. In these rotations, a post-graduate year (PGY) 2 or 3 resident leads a team of PGY1 interns and medical students to provide patient care under the supervision of faculty. Residents engage in multiple inpatient general medicine rotations per year. Faculty assess resident performance during these clinical rotations.

These clinical performance assessments use the Accreditation Council on Graduate Medical Education (ACGME)’s core competency framework [17]. While assessment tools vary by program, clinical performance assessments generally include items asking faculty to numerically rate resident performance and answer open-ended questions, providing narrative comments about resident performance. The evaluation tools used by three programs in our study asked about resident strengths and areas for improvement while the remaining queried about overall resident performance and competency-specific questions.

Clinical performance assessments collect and convey information about resident performance to programs and trainees [18]. Performance assessments serve a two-fold role of communicating information about trainee progress to programs and providing developmental feedback to residents to improve performance [19]. The ACGME requires programs to facilitate resident review of these assessments and encourage use the information to reinforce strengths and modify deficiencies [20].

Data for this study includes the written comments provided in clinical performance assessments; verbal feedback provided during rotations was not included. We use the terms performance feedback, qualitative assessment, and narrative comments to refer to the textual information provided in response to these open-ended questions from clinical performance assessments [21–23].

We also collected resident information including resident gender and post-graduate year (PGY). We used men and women gender designations as determined by participants’ professional gender identity as known to program directors or faculty members at each institution. Data was extracted from program management systems by program administrators at each site. Data de-identification was performed prior to analysis and included removing faculty and resident names and gendered pronouns from comment text.

### Qualitative content analysis

We performed a qualitative content analysis to explore themes in text comments provided in faculty assessments of resident performance [24–26]. We employed a multistage, multi-analyst approach that included familiarization and immersion with data, open coding of a subset of comments and generating a codebook through iterative coding and discussion and applying this coding framework to the larger sample of comments with regular discussion to refine codes. From this, we developed themes, or overarching concepts of meaning, iteratively using connections across data and refined though discussion.

We undertook multiple efforts to promote the trustworthiness of our findings. We analyzed comments from all evaluations collected to strengthen generalizability of our results [16]. Coding stages were performed multiple times with regular discussion and consensus building to maintain the quality and trustworthiness of the analysis. Coding was blinded to participants’ identity and gender. The coding team included 3 women (RK, ES, JK) IM physicians with experience in IM resident assessment (ES, JK, RK) and training in qualitative methods (RK). Coding was performed using coding software (MaxQDA).

We explored patterns in themes with resident gender and post graduate year. Specifically, we focused on whether there were differences and patterns in themes in comments about men and women residents, and how themes manifest in different years of training as residents progress from being a team member (PGY1) to a team leader (PGY2 and PGY3).

As a lens through which to explore perceptions of resident performance, we employed social role theory, which posits that one’s role is prescribed a socially defined set of traits and behaviors [27]. A prominent example of this is seen in communal qualities expected of women and agentic traits ascribed to men [28]. Through this lens, women are viewed as displaying a communal or interpersonal orientation characterized by concern for relationships with others, and are ascribed traits and behaviors that reflect this, such as kindness and nurturing. Men are viewed as having an agentic or self or task orientation characterized by agency and ability, and thus are ascribed traits and behaviors that reflect this, such as ambition and taking charge. Role conflict occurs when expectations around social roles diverge with what is expected in one’s professional role as physician or team leader. Expectations around gender roles may activate implicit bias in judgments about individuals, and individuals may experience role strain in the face of conflict.

Our analysis was informed by prior work in this same cohort which found gender-based differences in quantitative ratings and narrative comments provided in evaluations with women residents receiving positive but less specific comments than men residents, particularly in earlier years of training [4, 11].

#### Code Framework

Table 1 details themes developed in our analysis. Our findings are grouped into 2 broad themes: relationship-orientation and confidence and autonomy. Relationship-orientation refers to comment content that reinforces relationships with others and efforts to support and maintain these. Relationship-orientation includes subthemes of communal traits and attributes, communication and interpersonal skills, support role, and tone. Communal traits and attributes refers to traits and attributes that center the welfare of others over self. Communication and interpersonal skills refers to the ability to communicate and facilitate interactions with others. Support role refers to how the resident supports other team members. Tone refers to emotional tone or climate of the team and how the resident contributes to this. Confidence and autonomy refers to resident willingness and independence in decision-making and team leadership, and behaviors that convey confidence (delegating, assertiveness, and being vocal).

**Table 1:** Framework Used in Content Analysis from study of Gender and Thematic Differences in Narrative Comments of Internal Medicine Resident Performance

Theme	Subtheme	Description
Relationship Orientation	Communal Traits & Attributes	Use of traits and attributes that center the welfare of others over self
	Communication & Interpersonal Skills	Ability to communicate and facilitate interactions with others
	Support Role	Supporting others on the team
	Tone	Emotional tone or climate of the team
Confidence & Autonomy		Confidence in decision making and leadership and attributes and behaviors that convey confidence

We report themes in comments of men and women residents across years of training. Frequency of themes is reported as the proportion of evaluations containing comments in which the theme is present [29]. We present de-identified quotes to ensure anonymity of participants and sites [30].

Institutional Review Boards at each institution (Emory University, University of Louisville, University of Alabama Birmingham, University of California San Francisco, and Massachusetts General Hospital) exempted the study protocol. The study was deemed secondary research of existing data for which consent was not required.

## Results

Data included 3,383 evaluations with narrative comments, including 1834 assessments of 385 men residents (55.2%) and 1549 assessments of 313 women residents (44.8%). There were 1980 assessments with comments for PGY1 residents (58.5%), 823 assessments with comments for PGY2 residents (24.3%), and 580 assessments with comments for PGY3 residents (17.2%).

### Themes in qualitative assessments

Table 2 provides illustrative quotes for themes derived from our analysis.

**Table 2:** Themes in Qualitative Assessments about Residents from study of Gender and Thematic Differences in Narrative Comments of Internal Medicine Resident Performance

Theme & Subthemes	Illustrative Quotes about Women Residents	Illustrative Quotes about Men Residents
**Relationship Orientation**		
Traits & Attributes	*“Very warm and kind with patients.”* Competency Specific Comment, Woman PGY1 intern	*“They are enthusiastic, hard-working, warm and humble - which all allow them to function seamlessly in an intern role. They role-model their empathic approach to patients as well as highly developed exam and interview skills….”* Strengths and Areas for Improvement Comment, Man PGY1 intern
Communication & Interpersonal Skills	*“(First Name) has outstanding communication with patients and families. They are a true patient advocate and have outstanding professionalism and empathy...”* Strengths and Areas for Improvement Comment, Woman PGY3 resident *“(First Name) is excellent at getting things done and communicating effectively with team members. They are able to communicate their ideas and change people's minds without forcing it upon then, which will serve them well as they work with larger teams and leads others. I think their communication is one of their greatest strengths…”* Overall Comment, Woman PGY2 resident	*“(First Name) is an effective communicator. They have a pleasant affable nature which enabled them to form a strong rapport with their patients and work well with their colleagues. Their documentation was additionally spot on.”* Competency Specific Comment, Man PGY1 intern
Support Role	*“They are an effective team player, sharing the work, the learning and responsibility for the patients…”* Overall Comment, Woman PGY1 intern	*“They are also a great team member, and has stepped up multiple times (without complaint) to help other members of the team out…”* Overall Comment, Man PGY1 intern
	*“As far as leading the team I think, and more importantly their interns think, that they struck a fine balance between autonomy and supervision. Their interns felt that they were given space to think things through and advance their thoughts re a plan but that they were very supported and (First Name) was always aware of what was going on and available at a moment’s notice…”* Overall Comment, Woman PGY2 resident *“Their team members commented that they were very approachable and indeed multiple members of the team had approached them when there were issues in the team dynamics. (First Name) was effectively able to mitigate these situations and contribute to the personal growth of the members of their team...”* Overall Comment, Woman PGY2 resident	*“They encouraged autonomy in their interns but provided support for them in synthesizing the data they gathered into a thorough differential and thoughtful assessment and plan. They also role-modeled providing good patient care and solid communication skills.”* Competency Specific Comment, Man PGY2 resident
Tone	*“Wonderful attitude and sense of humor, a true joy to work with. Helps boost team morale...”* Overall Comment, Woman PGY3 resident *“The teams morale was incredibly high and this is largely due to their leadership and the tone they set…”* Overall Comment, Woman PGY2 resident *“They always maintained a calm demeanor, even in chaotic or stressful situations, which helped set a good tone for the team.”* Strengths and Areas for Improvement Comment, Woman PGY2 resident *“They are reserved by nature and this may at times make them seem less enthusiastic or confident than they truly are. I encourage them to work on projecting a more positive and enthusiastic demeanor, even if it feels like play-acting at first, as I believe it will greatly improve their therapeutic and professional relationships.”* Overall Comment, Woman PGY3 resident	*“Ensured the interns and students were given the mental learning space for team teaching in general. In terms of leadership, their calm demeanor and positive attitude set the tone for the team, which was an invaluable investment on their part….”* Strengths and Areas for Improvement Comment, Man PGY2 resident
**Confidence and Autonomy**		
	*“I encouraged them to more readily make commitments in their clinical decision making when discuss daily diagnostic and treatment plans for their patients.”* Competency Specific Comment, Woman PGY1 intern *“(First Name) is quiet but when spoken to or while participating in discussions they made valuable and relevant points. My suggestion to them was, you can and should speak up whether you agree or have an alternative view point. They did show they were indeed capable of good discussions and clearly this will impact their teaching skills positively.”* Overall Comment, Woman PGY1 intern	*“We discussed that they are a very strong intern with good medical knowledge and is great about always trying to enhance their medical knowledge. We discussed that they should continue to gain confidence with their assessments and plans and medical decision making.”* Overall Comments, Man PGY1 interns
	*“…They suffer from a bit of a lack of confidence which - catch 22 - stops them from putting themself out there sometimes. When they did make a real effort to come up with some teaching points on cases…They need to be pushed a bit more to keep this up as I think they has more teachable topics than they give themself credit for and it forces them to think through the topics more when they do this.”* Overall Comment, Women PGY2 resident *“Would continue to grow confidence in overall skills, and particularly in owning your authority as the team leader. Would also continue to experiment with ways to manage rounding time and patient care efficiently.“* Strengths and Areas for Improvement, Woman PGY2 resident *“My main feedback for (First Name) going into third year is to continue working on taking on more of a supervisory/leadership role. FName does an excellent job of being detail-oriented and helping the interns with their work load and overall development, and I think the next step is for them to spend more time searching the literature, teaching the interns/students, and directing the overall educational experience of the team (e.g. assigning subjects to look up/discuss as a team, sending relevant articles to the team, etc).”* Competency Specific Comment, Woman PGY2 resident *“Continue working on being a vocal team leader and directing the plans/presentations of junior team members – (First Name) has a soft-spoken approach, and they should be confident in presenting their plans and thoughts, as well as guiding how interns/students present their plans as well.”* Strengths and Areas for Improvement Comment, Woman PGY2 resident	*“Time management still have room to grow, as will their confidence about being more directive and authoritative at times.”* Strengths and Areas for Improvement Comment, Man PGY2 resident *“Has great knowledge and needs to have confidence with this.” Overall Comment, Man PGY3 resident*

Abbreviations: PGY, post graduate year

### Relationship orientation

Overall, 64.0% of trainee evaluation comments displayed communal or relationship orientation. This orientation was often expressed via citing communal traits and attributes that center the welfare of others over self. This included terms such as “kind,” “warm,” and “compassionate.”

In addition to attributes, this relationship orientation was also conveyed via a focus on residents’ relationship-oriented behaviors. This often manifested as a focus on communication and interpersonal skills with patients and team members. The following quote about a woman resident illustrates this emphasis on social skills and ability to facilitate interactions with the team.

“(First Name) is quite comfortable in the medical ‘team’ environment and their strong social skills make for smooth communication and interactions with all providers on the team.” Overall Comment, Woman PGY1 intern

In PGY1, this relationship orientation was seen in comments about men and women interns (62.5% comments of PGY1 women vs. 64.0% PGY1 men) as illustrated in the following comments.

“Their interpersonal skills and communication with families, and team members was superb…. They have a wonderful quality of kindness and compassion yet is efficient and able to prioritize multiple competing tasks with ease.” Overall Comment, Man PGY1 intern

“Extremely empathetic and compassionate, excellent communication with patients, families, and other team members. Respectful of all hospital staff, always exhibits a high level of integrity….” Strength and Areas for Improvement Comment, Woman PGY1 intern

Here the communal traits of both men and women interns are recognized and their communication skills with both patients and team members are praised. However, for the man, the communal focus of the comment is countered by also highlighting task-oriented skills and behaviors of efficiency and prioritizing.

Later in PGY2 and PGY3 when serving in the team leader role, this relationship orientation was persistent in comments about women residents both in emphasis and prevalence (69.8% comments of PGY2 and PGY3 women vs. comments of 60.9% PGY2 and PGY3 men). The following comment about a women PGY3 resident illustrates how multiple relationship building skills and attributes are accentuated which conveys these as the essential aspect of the resident’s performance.

“They are not just smart, kind, and hardworking, they are also empathic, warm, and approachable. They are the consummate team player and was always available to their interns, students, patients, and their families….” Overall Comment, Woman PGY3 resident

One notable relationship-oriented behavior of residents referenced in narrative comments was supporting and setting the tone for the team, seen in 36.4% of evaluations with comments. In PGY1, this collaborative work was most often framed as ‘being a team player’ and was seen in comments of both men and women interns (31.4% comments PGY1 women vs. 30.6% comments PGY1 men).

In PGY2 and PGY3, when residents are serving in the team leader role, this manifests as the way in which a resident supports the people on the team, namely the students and interns. The following quotes illustrate this support role and its actions.

“They actively looked out for the welfare of the interns and students and had a unique ability to deliver useful and constructive feedback in a positive supportive manner that helped them improve their performance. The students and interns learned a great deal under their tutelage in ways that will help them well beyond this rotation…” Strength and Areas for Improvement Comment, Woman PGY2 resident

“They were able to build-up the strengths of their team members and fostered rapport amongst team members….” Overall Comment Woman PGY2 resident

Here, the ways in which the residents support the people on the team are emphasized and framed as work the residents actively engage in via terms of supporting, fostering, building up, and advocating.

This support role work was referenced and emphasized in comments about women PGY2 and PGY3 residents more so than men residents (39.1% vs. 33.9%), as illustrated in the following quotes.

“Their commitment to their interns to have a safe, fun, and meaningful experience was obvious. They did this by being constantly supportive, focusing on teaching, and allowing the interns the space to make their decisions….” Overall Comment PGY2 Woman resident

“They enjoyed preparing interns and students for rounds by instruction and often supplemented my teaching points with relevant recent literature. Importantly, their calm leadership supported brand new interns as they effectively managed socially and medically complicated patients….” Overall Comment PGY3 Man resident

Both comments describe the residents’ support role for junior team members. In the comment about the man resident, this support role is conveyed in terms of tasks and actions as a teacher and a team leader. However, the comment about the woman resident frames the support role as a core characteristic of the woman resident’s performance via their ‘obvious commitment’ and ‘constant support’.

One further aspect of the relationship orientation theme was tone. This included the ways in which the resident established and sustained the emotional tone and climate of the team as a whole and how this affected others on the team. As seen in the following quotes, this most often appeared as praise in comments of PGY2 and PGY3 resident team leaders.

“They created a great learning environment that allowed their interns to grow and shine….” Overall Comment Woman PGY3 resident

“As a team leader, (First Name) sets a very comfortable and friendly tone for the group. The working environment under their guidance was one in which the rest of the team felt respected, heard, and wanted to come to work each day…”. Strength and Areas for Improvement Comment Woman PGY2 resident

“They created a highly effective learning environment [in which] all team members were comfortable exchanging ideas and learning from each other….” Strength and Areas for Improvement Comment Man PGY3 resident

As these quotes illustrate, tone is framed as something actively built and sustained by the resident rather than something that the resident simply contributes to via terms like ‘sets’ and ‘creates.’ In the comment about the man resident, tone is framed in terms of function as an environment that facilitated learning. In the comments about the woman resident, tone is framed as building a sense of community that focuses on the feelings and wellbeing of team members using terms such as comfort, friendly, respectful, and heard.

Supporting team members and creating the tone of the team was a persistent focal point of comments of women PGY2 and PGY3 residents compared to men residents, both in emphasis and prevalence.

### Confidence & autonomy

Another major theme noted across evaluation comments was resident confidence, autonomy, and independence. Overall, 25.7% of evaluation comments referenced confidence as either a strength or area for improvement. This was often framed as having room to grow in autonomy and confidence in their skills (14.7%). The following quotes illustrate this for PGY1 interns.

“Wonderful bedside manner, very patient and empathic. Excellent communicator with patients’ families. Would encourage to be more confident and decisive in medical decision-making.” Overall Comment, Women PGY1 intern

“(First Name) by nature is a bit quiet, and as a brand-new intern they were more hesitant than they might be otherwise, so sometimes they did not speak up even though they know the answer. I encouraged them to push themself outside of their comfort zone to do so as they are more capable than they give themself credit for.” Competency Specific Comment, Woman PGY1 intern

“I would challenge Dr. (Last Name) to balance their analysis with decisiveness. I think that this will come with experience, but I would challenge them to push themself toward independent decision making.” Competency Specific Comment, Man PGY1 intern

As demonstrated here, this theme encompassed perceptions of resident confidence and autonomy, and the agentic traits and behaviors that signal them such as decisiveness, committing to a plan, and being vocal. Often these comments were framed as encouragement and recommendations to adopt agentic behaviors that convey confidence, as illustrated in the following quote.

“Develop confidence in their clinical decision making, which is already sound, but could be presented with more confidence.” Area for Improvement, Women PGY2 resident

In PGY2 and PGY3, this theme often encompassed confidence in their role as team leader and actions that convey confidence as team leader such as delegating, being decisive, and directing others, as illustrated in the following quote.

“Dr. (Last Name) is very bright and hard working. They also are great at compassionate care. Dr. (Last Name) could improve in their leadership skills. They need to learn to delegate and not feel bad about it. Dr. (Last Name) could also improve on being decisive. They seem to exhaust themselves in a “paralysis by analysis” stage rather than acting on the knowledge that they possess. This unfortunately leads to inefficient patient care and Dr. (Last Name) being more tired than need be!”. Strengths and Areas for Improvement Comment, Woman PGY3 resident

Overall, confidence was noted more often in comments about women trainees than men (29.9% vs. 22.7%). For women, this emphasis on confidence was seen in PGY1 (29.9%) and persisted in PGY2 and PGY3 (26.9%).

Taken together, comments about women residents at times referenced both relationship building and confidence themes, as illustrated in the following quotes. For women, this manifested as praise for communal attributes, while also recommending autonomy, confidence, and behaviors that convey these agentic attributes.

“(First Name) has a strong clinical instinct that they can grow into trusting a little more and be decisive and directive in communication both to their attending as well as the ones they are managing. They can combine the compassionate demeanor with being a bit more directive. They are sometimes open to supporting individuals so much that they must balance the needs of patient care, and realities of ward team….” Strength and Areas for Improvement Comment, Woman PGY2 resident

“(First Name) has the potential to be a star third year. They could be more aggressive in their teaching role on rounds. Their management of the two interns was excellent, but now that they have got some experience with interns needing attention, they could be more aggressive with interventions. They have a very kind and thoughtful approach to interpersonal interactions but needs to feel more comfortable laying out expectations and holding people accountable…”. Area for Improvement, Woman PGY3 resident

Here, acknowledgement of the residents’ supportive approach to team leadership was tempered with multiple recommendations to adopt more agentic leadership behaviors (i.e., being decisive, directing, being aggressive, setting expectations, holding accountable). In contrast, these agentic leadership skills were often cited as strengths for men resident team leaders, as illustrated in the following comment.

“(First Name) has committed themself to being a superb team leader and is clearly moving toward that goal. As I already implied, their primary method is to lead by example. They were able to seamlessly delegate responsibility to the interns, allowing them to take the helm without ever losing sight of where the ship was heading. Furthermore, they were able to do this in subtle fashion which provided lubrication, rather than unwanted friction, to the team function….” Overall Comment Man PGY2 resident

## Discussion

In this multisite study of narrative comments in performance assessments, there were thematic differences in comments received by men and women residents and the ways in which these themes manifested within comments shifted with time in training. We found comments about women resident team leaders had a persistent relationship-orientation which emphasized communal attributes, communication and interpersonal skills, role in supporting others, and establishing the tone for the team. Throughout training comments about women residents referenced confidence and recommended behaviors that convey confidence in decision making and team leadership.

A relationship-orientation to narrative comments is consistent with prior studies that found certain communal terms were more common in qualitative assessments of women compared to men in both undergraduate and graduate medical education [8–10]. The current findings suggest that the communal orientation is present not only in the attributes ascribed to women residents, but also the skills and behaviors referenced in assessment feedback including communication, interpersonal skills, supporting others and setting the tone for the team.

A novel aspect of our analysis is the way in which the relationship orientation of comments is variably expressed and emphasized across post graduate year. Comments of both men and women interns often cited communal traits and behaviors. However, in PGY2 and PGY3 when serving in the team leadership role, comments about women residents had a persistent focus on relationship-building skills and attributes compared to men. These findings suggest that the intern role, more so than the senior resident team leader role, is seen as embracing communal attributes and skills. These differences in role expectations may then contribute to gendered differences in comments. This supports prior study of qualitative assessments of emergency medicine (EM) residents which posited that differences in expectations of intern and resident roles contribute to gendered differences in assessment [31].

Comments about women resident team leaders emphasized supporting team members and creating the tone of the team, in essence building a sense of community for the team as the team leader. These are characteristic of the communal role ascribed to women’s gender roles.

We also noted confidence and autonomy to be a theme across comments received by women residents. This is consistent with studies of assessment feedback for both EM and IM residents which found that women received comments that more often referenced confidence [13, 14]. While references to under-confidence were most common, the mention of appropriate confidence may also imply expectations around confidence.

Confidence involves both action and certainty [32]. The attributes of a confident team leader – decisiveness, assertiveness, delegating, directing – are characteristic of the agentic attributes more often ascribed to men’s gender roles. We found that comments about women resident team leaders often referenced confidence as team leader and actions that convey confidence. These findings align with a study of narrative comments provided to anesthesia residents which found that comments about women less often referenced agentic traits and behaviors overall and that these agentic traits and behaviors were often reserved for more senior residents [33].

At times women resident team leaders were commended for their relationship building skills and attributes that align with what is expected based on gender role while also encouraged to display more agentic attributes and behaviors. This suggests that women residents are coping with the ‘double bind’ that women leaders face. The ‘double bind’ refers to the way in which women leaders must be seen as having communal qualities to be seen as likeable while also displaying agentic traits to be seen as effective or competent leaders [34, 35]. Our finding that women residents are praised for communal behaviors that align with gender role expectations but also encouraged to adopt behaviors that convey confidence may indicate that women residents may be held to a standard that requires them to navigate this balance in their leadership roles during residency training. The act of threading this needle may be reflected in performance and performance assessments. Evidence suggests that women residents experience strain when their professional role requires them to act counter to gender-based normative behaviors and this may impact performance [36].

These findings have important implications for learners. Qualitative assessments serve as developmental feedback about learner performance to learners and programs. Prior work in this same dataset found gender-based differences in the specificity and valence of narrative comments with women receiving praising but less specific assessment feedback [11]. Taken together, the persistent relationship orientation in comments about women residents while receiving praising but less detailed feedback suggests that these communal behaviors and skills are highlighted at the expense of others. This suggests a lost opportunity for well-rounded assessment feedback, which may disadvantage women residents. Gendered differences in narrative comments may reflect and further reinforce gendered expectations for women residents, especially in the team leader role.

Limitations of this work include the retrospective, cross-sectional nature of the data which does not allow for assessing differences within residents over time. Due to limitations in our sample, this study does not address the experiences of those identifying as gender nonbinary. In addition, it is likely that other social factors, such as race and class, play a role in the disparities we found.

Overall, these findings suggest thematic differences in qualitative assessments men and women residents receive in training. Comments about women resident team leaders emphasize relationship building and urge confidence and actions that convey confidence as team leader. The attention to communal skills suggests gendered expectations for women resident team leaders and a lost opportunity for well-rounded feedback to the disadvantage of women residents. These findings may inform interventions to promote equitable assessment, such as encouraging assessment feedback across the core competencies.

## Data Availability

Datasets generated and analyzed during the current study are not publicly available due to restrictions on sharing assessment data. For further inquiry regarding the study, contact the corresponding author.
