# Correction: Exploring gender and thematic differences in qualitative assessments of internal medicine resident performance

**DOI:** 10.1186/s12909-023-05000-x

**Published:** 2024-01-25

**Authors:** Robin Klein, Erin D. Snyder, Jennifer Koch, Anna Volerman, Sarah Alba-Nguyen, Katherine A Julian, Vanessa Thompson, Nneka N Ufere, Sherri-Ann M Burnett-Bowie, Anshul Kumar, Bobbie Ann Adair White, Yoon Soo Park, Kerri Palamara

**Affiliations:** 1 Emory University School of Medicine, Atlanta, GA USA; 2 Department of Medicine, Division of General Internal Medicine, University of Alabama Birmingham School of Medicine, Birmingham, AL USA; 3 Department of Medicine, University of Louisville, Louisville, KY USA; 4 Departments of Medicine and Pediatrics, University of Chicago, Chicago, IL USA; 5 Department of Medicine, Division of Hospital Medicine, University of California, San Francisco, San Francisco, CA USA; 6 Department of Medicine, Division of General Internal Medicine, University of California, San Francisco, San Francisco, CA USA; 7 Department of Medicine, Division of Gastroenterology, Massachusetts General Hospital, Boston, MA USA; 8 Department of Medicine, Endocrine Division, Massachusetts General Hospital, Boston, MA USA; 9 Massachusetts General Hospital Institute of Health Professions, Boston, MA USA; 10 Department of Medical Education, University of Illinois, Chicago, IL USA; 11 Department of Medicine, Massachusetts General Hospital, Boston, MA USA

**Correction: BMC Medical Education (2023) 23:932**

10.1186/s12909-023-04917-7

Following publication of the original article [1], we have been informed about formatting errors on quotes and also in Table 2.

The original article has been corrected.
